# GRA86 Is a Novel Dense Granule Protein Important for Virulence and Bradyzoite Differentiation in *Toxoplasma gondii*

**DOI:** 10.3390/ani15172591

**Published:** 2025-09-03

**Authors:** Xiao-Nan Zheng, Jing Li, Xin-Sheng Lu, Hany M. Elsheikha, Xing-Quan Zhu

**Affiliations:** 1 Laboratory of Parasitic Diseases, College of Veterinary Medicine, Shanxi Agricultural University, Jinzhong 030801, China; lijing1127x@163.com (J.L.); luxinsheng2024@126.com (X.-S.L.); 2 Faculty of Medicine and Health Sciences, School of Veterinary Medicine and Science, University of Nottingham, Sutton Bonington Campus, Loughborough LE12 5RD, UK; hany.elsheikha@nottingham.ac.uk

**Keywords:** bradyzoite differentiation, dense granule protein, GRA86, subcellular localization, *Toxoplasma gondii*, toxoplasmosis, virulence

## Abstract

*Toxoplasma gondii* is a globally prevalent parasite capable of establishing lifelong infections, which can have severe consequences in immunocompromised individuals and developing fetuses. GRAs are essential secretory effectors that facilitate nutrient acquisition, modulate host immune responses, and support intracellular survival. In this study, we characterized four putative GRAs (GRA85–88) that co-localize with GRA12 in both tachyzoite and bradyzoite stages. Using CRISPR-Cas9-mediated homologous recombination, we successfully generated knockout strains in both type I RH and type II Pru backgrounds. Phenotypic analysis revealed that GRA85, GRA87, and GRA88 were not individually required for parasite replication, invasion, or virulence. However, deletion of *gra86* (PruΔ*gra86*) resulted in a significant reduction in virulence and fewer brain cysts in chronically infected mice, although in vitro growth remained unaffected. Transcriptomic profiling of PruΔ*gra86* revealed downregulation of bradyzoite–related genes and upregulation of GRAs involved in host interaction. Additionally, in vitro differentiation assays showed impaired bradyzoite development in the absence of GRA86. These findings from murine models and in vitro phenotypic assays highlight GRA86 as a regulator of chronic infection and stage conversion, positioning it as an important player in *T. gondii* pathogenesis and a promising target for therapeutic intervention.

## Simple Summary

*Toxoplasma gondii* is a ubiquitous intracellular parasite that infects nearly all warm-blooded animals, including humans. It relies on secreted effectors to manipulate its environment and ensure survival. Key to its ability to establish and maintain infection is various secretory effectors, particularly dense granule proteins (GRAs). Although more than 100 GRAs have been identified, many remain poorly characterized. In this study, we examined the subcellular localization and biological roles of four predicted GRAs (GRA85–88). Immunofluorescence analysis of endogenously tagged strains revealed that these proteins localize to the parasitophorous vacuole in tachyzoites and to the cyst matrix in bradyzoites. Using CRISPR-Cas9 technology, we generated knockout strains for each gene, and phenotypic assays demonstrated that GRA86 is important for parasite virulence and brain cyst formation during chronic infection with the type II Pru strain, although it is not required for in vitro growth. In contrast, GRA85, GRA87, and GRA88 were individually dispensable for both in vitro and in vivo fitness. Further transcriptional profiling and in vitro assays suggested that GRA86 plays a pivotal role in bradyzoite differentiation. Together, these findings highlight the significant role of GRA86 in chronic infection and stage conversion, positioning it as a promising target for future therapeutic interventions.

## 1. Introduction

*Toxoplasma gondii*, the causative agent of toxoplasmosis, is a ubiquitous parasite that infects almost all warm-blooded animals, including humans, with about one-third of the global human population infected [1,2]. While primary infection is often asymptomatic in immunocompetent individuals, it can cause serious health complications in immunocompromised patients and developing fetuses [2,3,4]. Congenital toxoplasmosis represents a major global health burden, with an overall prevalence of acute *T. gondii* infection in 1.1% of pregnant women. This infection is responsible for stillbirths, neurological damage, and visual impairment in survivors [2,3,4,5,6]. Transmission of *T. gondii* commonly occurs via consumption of raw or undercooked meat containing cysts with bradyzoites, or unwashed vegetables and fruits contaminated with oocysts containing sporozoites [7]. Once inside the host, these forms differentiate into tachyzoites, the rapidly proliferating stage that causes acute infection and tissue damage [8,9]. In response to immune pressure, tachyzoites convert into bradyzoites, which encyst in host tissues, establishing a chronic infection that can persist for years [9,10]. In immunocompromised individuals, bradyzoites can be reactivated, converting back into tachyzoites, which lead to severe or fatal disease [9]. Current therapies are limited by their side effects and failure to eliminate tissue cysts, highlighting the urgent need for novel therapeutic targets and vaccine candidates.

The survival and persistence of *T. gondii* within its host cells depend on a complex array of secretory effectors, including micronemal proteins (MICs), rhoptry proteins (ROPs), and dense granule proteins (GRAs). MICs are involved in initial host cell attachment, while ROPs and rhoptry neck proteins (RONs) are crucial for the formation of the parasitophorous vacuole (PV), a specialized membrane-enclosed niche for parasite replication [11,12]. Following invasion, GRAs are secreted into the PV and then remain there, associate with the intravacuolar network (IVN), integrate into the parasitophorous vacuole membrane (PVM), or are exported to the host cytoplasm or nucleus [12,13]. These GRAs modulate key cell processes such as nutrient acquisition, trafficking of effector proteins, and immune response modulation [11,12,13]. The PVM, which serves as the host–parasite interface, is a primary target for host immune defense. To circumvent immune recognition, *T. gondii* deploys a variety of effector proteins, such as ROP5, ROP17, and ROP18, which inhibit host immunity-related GTPases (IRGs) to prevent PV destruction [11,14,15,16,17]. Several GRAs, including GRA16 and GRA15, play pivotal roles in immune evasion and host cell manipulation after precise trafficking to final destinations by proteins, such as MYR1, GRA44, and GRA45, further contributing to parasite survival [18,19,20,21,22,23,24,25,26,27,28,29].

In chronic infection, tachyzoites differentiate into bradyzoites that replicate slowly within tissue cysts, primarily in the brain and skeletal muscles. This stage transition is essential for maintaining long-term infection, and specific GRAs are critical for both cyst formation and persistence. For example, GRA4, GRA6, and GRA12 associate with the IVN and relocalize to the developing cyst wall during early differentiation, while other GRAs, such as PVM-associated GRA5 and GRA7, assist in cyst maturation [30,31,32]. In addition, bradyzoite-specific GRAs, including GRA55 and GRA59, are essential for cyst formation, with the deletion of certain GRAs, such as CST2 and GRA55, leading to a significant reduction in cyst burden in vivo [33,34]. Previously, we reported that GRA76, which is more highly expressed in tachyzoites than in bradyzoites, is crucial for cyst formation [35]. Moreover, GRA12, GRA47, and GRA72 have been shown to play important roles in establishing and maintaining brain cysts in animal models [36,37,38].

Despite the identification of over 100 GRAs, many remain poorly characterized, particularly those predicted through advanced proteomic approaches such as hyperLOPIT (hyperplexed Localization of Organelle Proteins by Isotopic Tagging). Understanding the functions of these putative GRAs is essential for unraveling the mechanisms underlying *T. gondii* pathogenesis and for identifying potential therapeutic targets. The three major clonal lineages of *T. gondii* (types I, II, and III) exhibit distinct virulence profiles in murine models: Type I strains are highly virulent, type II strains exhibit intermediate virulence influenced by host genetics, and type III strains are avirulent [39,40,41]. The RH (type I), Pru (type II), and VEG (type III) strains serve as key reference models in experimental studies.

In this study, we focus on four predicted GRAs (GRA85–GRA88) to investigate their roles in parasite growth, virulence, and chronic infection. By using CRISPR-Cas9 technology to generate knockout strains in both type I RH and type II Pru backgrounds, we aim to provide insight into the functions of these putative GRAs and their potential as targets for therapeutic intervention.

## 2. Materials and Methods

### 2.1. Mice

Eight-week-old female Kunming mice were purchased from Beijing Sibeifu Biotechnology Co., Ltd. (Beijing, China) and maintained under specific pathogen-free conditions in an environmentally controlled setting (12-h light/dark cycle, 22 °C, 50–60% humidity). Mice had ad libitum access to sterilized food and water [37,42]. Prior to experimental infection, mice were acclimated for at least one week. Six mice were included in each infection group. Mice were euthanized promptly upon reaching the humane endpoint (20% body weight loss). All procedures were carried out with careful consideration to minimize animal suffering.

### 2.2. T. gondii Strains and Cell Culture

The *T. gondii* strains used in this study included RHΔ*ku80* (type I, referred to as RH) and PruΔ*ku80* (type II, referred to as Pru), both maintained in confluent monolayers of human foreskin fibroblasts (HFFs, ATCC SCRC-1041). HFFs were cultured in Dulbecco’s Modified Eagle Medium (DMEM, Gibco, Suzhou, China) supplemented with 10% fetal bovine serum (FBS, Gibco, Melbourne, VIC, Australia), 10 mM HEPES (pH 7.2, Solarbio, Beijing, China), and 100 U/mL penicillin and 100 µg/mL streptomycin (Solarbio, Beijing, China), at 37 °C in 5% CO_2_ [36,43,44]. For tachyzoite culture and transfection, 2% FBS was used. Infected HFFs were passed through a 27-gauge needle and filtered through a 3 µm membrane to isolate tachyzoites.

### 2.3. Construction of Transgenic Parasite Strains

For C-terminal endogenous tagging, a CRISPR-Cas9 plasmid targeting the 3′ untranslated region (UTR) of the gene was constructed by replacing the original sgRNA in the pSAG1::CAS9-U6-sgUPRT plasmid with a custom sgRNA targeting the stop codon region of each novel GRA gene [44]. This plasmid was co-transfected into RH tachyzoites along with PCR amplicons (~42 bp homology arms) encoding a 6× hemagglutinin (6HA) tag and a dihydrofolate reductase (DHFR) resistance cassette. Positive clones were selected using 3 μM pyrimethamine and confirmed by PCR and sequencing.

Gene knockouts were generated using CRISPR-Cas9–mediated homologous recombination, where the coding sequences of the GRAs were replaced with a homologous fragment containing a DHFR selectable marker flanked by the respective GRA gene’s upstream and downstream regions, as previously described [45]. Plasmids were constructed by assembling DHFR selection markers with a pUC19 backbone and ~1 kb homology arms corresponding to the 5′ and 3′ UTRs of each gene. The linearized donor fragments and CRISPR-Cas9 plasmids targeting the gene’s coding region were co-transfected into RH tachyzoites. Drug-resistant clones were selected and validated by PCRs. Primer sequences used for the construction and validation of transgenic strains are listed in Supplementary Table S1.

### 2.4. Bradyzoite Conversion

Bradyzoite differentiation of wild-type *T. gondii* RH or Pru strains (control groups) and gene knockout strains (experimental groups) was induced by alkaline stress, as previously described [35,46]. Briefly, RH and Pru parental tachyzoites were allowed to infect HFF monolayers at a multiplicity of infection (MOI) of 0.5 for 2 h and 4 h, respectively, in medium at pH 7.4. To induce alkaline stress, the medium was replaced with bradyzoite induction medium (pH 8.2), supplemented with 2% FBS. For the RH (type I) parental strains, the medium was replaced daily with fresh induction medium; for the Pru (type II) parental strains, the medium remained unchanged. Differentiation was carried out at 37 °C in ambient CO_2_ for 48 h under alkaline conditions. Bradyzoite cysts were identified by indirect immunofluorescence using FITC-conjugated *Dolichos biflorus* agglutinin (DBA, Vectorlabs, Burlingame, CA, USA), which specifically binds N-acetylgalactosamine on the cyst wall [32]. Parasites were co-stained with rabbit anti-IMC1 (1:500, available in our laboratory) and Alexa Fluor 594-conjugated goat anti-rabbit IgG (1:500, Thermo Fisher Scientific, Waltham, MA, USA).

### 2.5. Indirect Immunofluorescence Analysis (IFA)

HFFs infected with parasites at a MOI of 0.5 were fixed in 4% paraformaldehyde (PFA) for 20 min, permeabilized with 0.2% Triton X-100 for 15 min, and blocked with 3% BSA. Primary antibodies were incubated for 2 h at 37 °C, followed by incubation with secondary antibodies for 1 h at 37 °C. Between each step, samples were washed with PBS. Images were captured using a Nikon Eclipse Ti2-U fluorescence microscope at 100× magnification.

### 2.6. Subcellular Localization of Novel GRAs

HA-tagged GRAs were detected using mouse anti-HA (1:500, Thermo Fisher Scientific, Waltham, MA, USA) and Alexa Fluor 594-conjugated goat anti-mouse IgG (1:500, Thermo Fisher Scientific, Waltham, MA, USA). GRA12, a marker for dense granules, was visualized using rabbit anti-GRA12 (1:500, available in our laboratory) and Alexa Fluor 488 or 647–conjugated secondary donkey anti-rabbit IgG antibodies (1:500, Thermo Fisher Scientific, Waltham, MA, USA). Differentiated bradyzoite-encysted cysts were identified using DBA, and nuclei were counterstained with DAPI (1:500, Thermo Fisher Scientific, Waltham, MA, USA).

### 2.7. Plaque Assays

Freshly egressed tachyzoites (500 per well) were added to 12-well plates containing monolayers of approximately 2 × 10^5^ HFF cells and incubated for 7 or 15 days. After incubation, cells were washed, fixed with 4% PFA, and stained with 0.2% crystal violet (Solarbio, Beijing, China) for 20 min. Plaques were imaged, and their sizes were analyzed using ImageJ software (v1.53a, NIH, Bethesda, MD, USA).

### 2.8. Invasion Assays

To assess the impact of individual gene deletions on invasion, HFF monolayers were infected with purified tachyzoites of wild-type RH strain or gene knockout RHΔ*gra* strains at a MOI of 3 for 1 h. After gentle washing, IFA was performed. Extracellular parasites were labeled with mouse anti-SAG1 (1:500, Thermo Fisher Scientific, Waltham, MA, USA) and Alexa Fluor 488-conjugated goat anti-mouse IgG (1:500, Thermo Fisher Scientific, Waltham, MA, USA) before permeabilization. Total parasites (both intra- and extracellular) were then permeabilized and labeled with rabbit anti-IMC1 (1:500) and Alexa Fluor 594-conjugated goat anti-rabbit IgG. One hundred parasites per replicate were counted to calculate invasion efficiency [35,45].

### 2.9. Intracellular Replication and Egress Assays

To assess replication, tachyzoites of RH and RHΔ*gra* strains were added to HFF monolayers at a MOI of 0.5. One hour post-infection, uninvaded parasites were removed by washing with fresh medium. After 24 h, cells were fixed, permeabilized, and stained with anti-IMC1 and Alexa Fluor 488. The number of tachyzoites per PV was counted in at least 100 PVs per sample [47].

Egress is crucial for parasite dissemination within the host. To examine the role of GRAs in egress, tachyzoites of RH and RHΔ*gra* strains were allowed to replicate for 36 h, by which time most PVs contained at least 16 parasites. Following treatment with calcium ionophore A23187 (Sigma, Saint Louis, MO, USA) for 2 min, samples were fixed, and the number of PVs with egressed and non-egressed tachyzoites was quantified to determine the percentage of egressed PVs (n ≥ 200 per group) [48].

### 2.10. Virulence Assessment

Mice were intraperitoneally infected with freshly egressed tachyzoites from wild-type (control group) or gene knockout strains (experimental group, with viability confirmed via plaque assay) at an infective dose of 1 × 10^2^ tachyzoites for type I strains, and 2 × 10^2^ or 5 × 10^4^ for type II strains [46,49]. Mice were monitored for 30 days for clinical symptoms and humane endpoint. Serum samples were collected for ELISA-based detection of total IgG, IgG1, and IgG2a anti-*T. gondii* antibodies [42,50]. To assess cyst burden, brains from surviving mice were harvested, homogenized, and analyzed under a microscope, as described previously [35,36,38]. All animal procedures were approved by the Institutional Animal Care and Use Committee of Shanxi Agricultural University (Approval No.: SXAU-EAW-2021XM121001) and adhered to Chinese ethical guidelines for animal experimentation.

### 2.11. RNA Sequencing and Real-Time Quantitative qPCR (RT-qPCR)

Pru and PruΔ*gra86* tachyzoites were used to infect HFF monolayers at a MOI of 5 for 48 h. Heavily infected cultures were harvested for total RNA extraction using TRIzol, followed by DNase treatment. RNA quantity and quality were assessed using a NanoDrop and the Agilent 2100 Bioanalyzer, respectively. RNA-seq libraries were prepared and sequenced using the BGI-AEQ platform (Shenzhen, China) [35]. Reads were quality-trimmed with SOAPnuke and aligned to the *T. gondii* ME49 genome (https://toxodb.org (accessed on 18 December 2024) using HISAT [51]. Differential expression analysis was performed using RSEM (v1.3.1) and DESeq2 (v1.4.5) [52,53]. In DESeq2, gene-specific dispersion was estimated using an empirical Bayes approach: (i) raw dispersion estimates were calculated per gene using maximum likelihood, (ii) these estimates were fitted to a mean-dispersion trend model across all genes, and (iii) final dispersion values were shrunk toward the fitted prior to improve robustness in small-sample analyses [53]. To control the false discovery rate (FDR) in multiple tests, raw *p*-values were adjusted using the Benjamini–Hochberg procedure, generating adjusted *p*-values (denoted as *Q* values) [53]. Genes with log_2_ fold change ≥ 1 or ≤ −1 and *Q* value ≤ 0.05 were considered differentially expressed. RT-qPCR was performed using a LightCycler 480 (Roche) to validate the expression of 10 upregulated genes and 10 downregulated bradyzoite-associated genes in intracellular parasites (MOI 5) harvested from infected HFF monolayers [54]. β-tubulin (TGME49_266960) was used as the internal control [54], with the Pru strain as the control group and the PruΔ*gra86* strain as the experimental group. Primer sequences are listed in Supplementary Table S2.

### 2.12. Statistical Analysis

All data are presented as the mean ± standard deviation (SD) from three independent experiments. Statistical analysis was conducted using GraphPad Prism v9.0. Differences between two groups were assessed using unpaired two-tailed Student’s *t*-tests, while one-way ANOVA was used for comparisons among three or more groups. A *p*-value of ≤ 0.05 was considered statistically significant.

## 3. Results

### 3.1. Expression of Four Newly Identified Toxoplasma Dense Granule Proteins in Both Tachyzoite and Bradyzoite Stages

**Figure 1:**
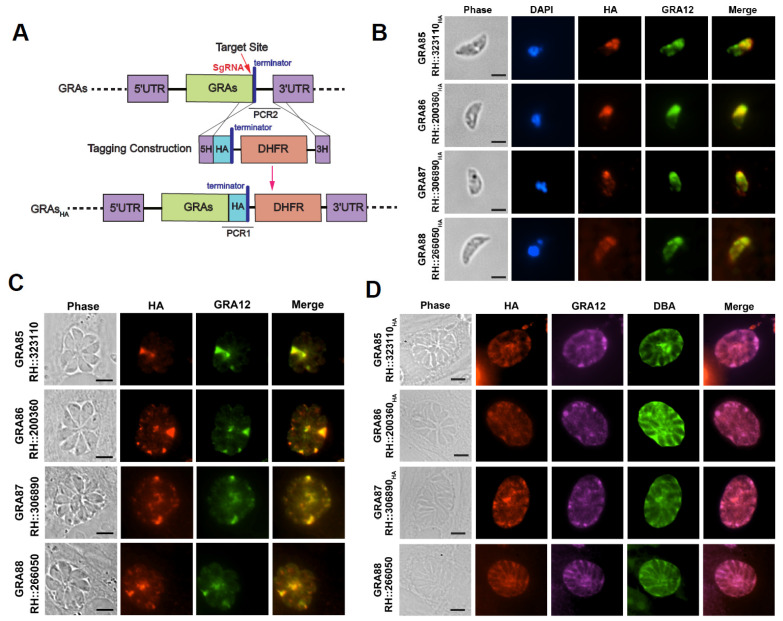
Localization of newly identified *Toxoplasma* dense granule proteins (GRA85–88). (**A**) Schematic representation of the CRISPR-Cas9-mediated endogenous C-terminal 6HA tagging of GRAs. (**B**) IFA analysis of extracellular RH tachyzoites showing partial co-localization of the novel GRAs (red, HA) with the dense granule marker GRA12 (green). Nuclei are stained with DAPI (blue). Scale bar: 2.5 μm. (**C**) IFA of intracellular tachyzoites within HFF cells demonstrating co-localization or partial co-localization of GRAs (red, HA) with GRA12 (green) in the PV. Scale bar: 5 μm. (**D**) IFA of in vitro differentiated bradyzoites under alkaline stress conditions showing co-localization of GRA85–88 (red, HA) with GRA12 (magenta) and the cyst wall marker FITC-DBA (green). Scale bar: 5 μm.

Four hypothetical proteins were previously predicted to localize to dense granules based on hyperLOPIT data (Table 1) [55]. To confirm their classification as GRAs, we endogenously tagged these proteins at the C-terminus with a 6HA epitope using CRISPR-Cas9-mediated homologous recombination (Figure 1A). Successful tagging was verified by PCR and sequencing (Figure S1).

Subcellular localization was assessed using IFA, with GRA12, a known dense granule marker, as a reference [36,38]. In extracellular RH tachyzoites, all four proteins, TGME49_323110, TGME49_200360, TGME49_306890, and TGME49_266050, partially co-localized with GRA12, supporting their classification as GRAs (Figure 1B). In intracellular tachyzoites, these proteins were predominantly secreted into the PV, where they also partially overlapped with GRA12 (Figure 1C).

To investigate their expression during the bradyzoite stage, we induced cyst differentiation in vitro using alkaline stress. In the resulting cysts, all four proteins localized to the cyst matrix or cyst wall, co-localizing with both GRA12 and FITC-conjugated DBA, a marker for the cyst wall (Figure 1D). This suggests that these proteins may play a role in cyst development or maintenance.

Based on these localization and expression patterns, we designated these proteins as GRA85 (TGME49_323110), GRA86 (TGME49_200360), GRA87 (TGME49_306890), and GRA88 (TGME49_266050). These results confirm that GRA85–88 are novel members of the GRA family and are expressed in both the tachyzoite and bradyzoite stages of *T. gondii*.

### 3.2. Successful Construction of Four Novel GRA Gene Knockout Strains

**Figure 2:**
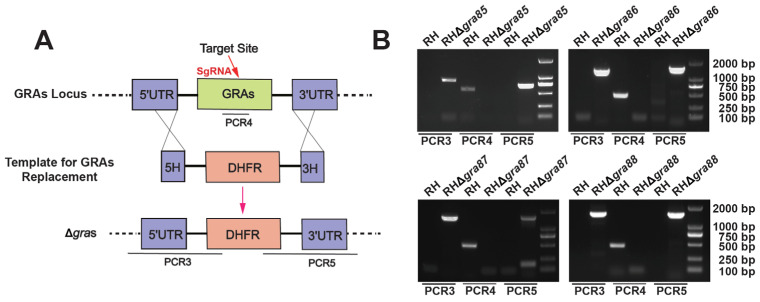
Construction and validation of RHΔ*gra* knockout strains. (**A**) Schematic representation of the CRISPR-Cas9-mediated homologous recombination strategy for gene knockout of the genes. (**B**) PCR validation of RHΔ*gra* knockout strains. The recombination of the 5′ and 3′ untranslated regions (UTRs) with the DHFR cassette was confirmed using PCR3 and PCR5 primers. The absence of GRA coding sequences in the knockout strains was validated using PCR4.

We successfully disrupted each GRA gene in both RH and Pru strains using CRISPR-Cas9-mediated homologous recombination (Figure 2A). PCR analysis (Figure 2B) confirmed the knockout of the GRA genes. Specifically, primers PCR3 and PCR5 amplified the expected DHFR fragment and flanking UTR regions in the RHΔ*gra* strains, but not in the wild-type RH. In contrast, PCR4, which targets a ~400 bp region of the GRA coding sequences, amplified products only in the wild-type RH, confirming the absence of the GRA coding sequences in the knockout strains. These results validate the successful generation of the RHΔ*gra85–88* knockout strains.

### 3.3. Four Novel GRAs Are Dispensable for In Vitro and In Vivo Fitness of the RH Strain

**Figure 3:**
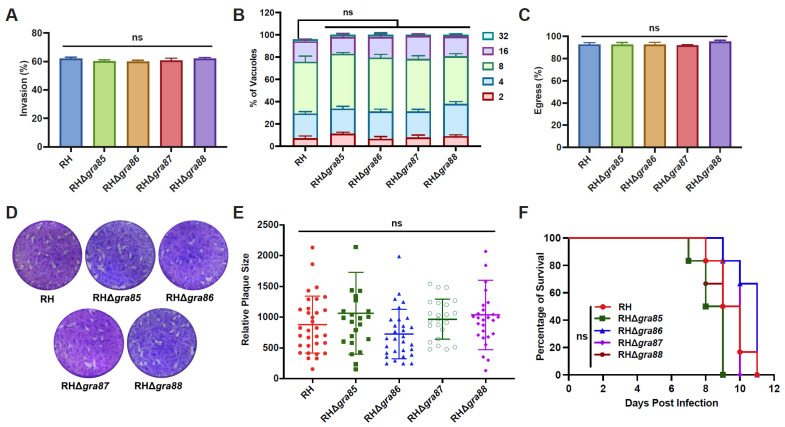
GRA85–88 are dispensable for the fitness and virulence of the *T. gondii* RH strain. (**A**) Invasion efficiency of RHΔ*gra* strains compared to wild-type RH. (**B**) Intravacuolar replication of the indicated strains, assessed by counting the number of tachyzoites in PVs at 24 h post-infection. (**C**) Egress efficiency of the indicated strains following treatment with calcium ionophore. (**D**) Representative plaques formed by RH and RHΔ*gra* strains on HFF monolayers at 7 days post-infection. (**E**) Quantification of relative plaque sizes. (**F**) Survival curves of Kunming mice infected with 100 tachyzoites of RH or RHΔ*gra* strains (6 mice per group). Data are presented as means ± SD from three independent experiments. ns indicates no significant difference.

The lytic cycle of *T. gondii* tachyzoites in vitro involves invasion, intracellular replication, and egress. Our results show that the disruption of *gra85*, *gra86*, *gra87*, or *gra88* did not affect the invasion efficiency (Figure 3A). Twenty-four hours post-infection in HFFs, the majority of PVs contained eight tachyzoites across all strains, with no significant differences in replication rates observed between the RHΔ*gra* and wild-type RH strains (Figure 3B). Moreover, egress efficiency was comparable between the knockout and wild-type strains (Figure 3C).

Plaque assays were performed to assess the overall impact of GRA disruption on the in vitro lytic cycle. Representative plaque images and quantitative analysis revealed no significant differences in plaque size between the knockout and wild-type strains (Figure 3D,E), indicating that GRA85–88 are dispensable for in vitro growth.

To investigate the role of GRA85–88 in vivo, Kunming mice were intraperitoneally injected with 100 tachyzoites of either RH or RHΔ*gra* strains. All infected mice reached their humane endpoint within 8.5 to 11 days, with no significant difference in survival time between the groups (Figure 3F). These results demonstrate that GRA85–88 are not essential for the fitness or virulence of the *T. gondii* RH strain, both in vitro and in vivo.

### 3.4. Knockout of the GRA86 Gene Reduces Pru Strain Virulence and Brain Cyst Burden In Vivo

**Figure 4:**
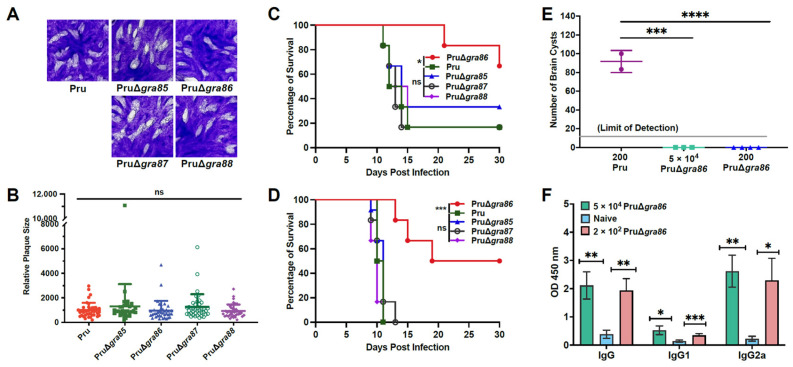
GRA86 is important for the virulence of *T. gondii* Pru strain. (**A**) Representative plaque images from wild-type Pru and PruΔ*gra* strains. (**B**) Quantification of relative plaque areas. (**C**) Survival curves of Kunming mice infected with 2 × 10^2^ tachyzoites. (**D**) Survival curves of mice infected with 5 × 10^4^ tachyzoites. (**E**) Brain cyst burden in surviving mice 30 days post-infection. (**F**) Serum levels of IgG, IgG1, and IgG2a antibodies in surviving mice at 30 days post-infection. Data are presented as means ± SD. Level of significance are *, *p* ≤ 0.05; **, *p* ≤ 0.01; ***, *p* ≤ 0.001; ****, *p* ≤ 0.0001; ns, not significant.

Gene knockouts of *gra85–88* in the type II *T. gondii* Pru strain (PruΔ*gra85–88*) were successfully generated (Figure S2). Plaque assays revealed no significant differences in plaque size between wild-type Pru and the PruΔ*gra* strains, indicating that the individual deletions of *gra85–88* do not affect in vitro growth of the type II parasites (Figure 4A,B).

In vivo assays showed that the survival rates of mice infected with PruΔ*gra85*, PruΔ*gra87*, and PruΔ*gra88* were similar to those infected with wild-type Pru, with 16.67–33.33% survival at 30 days post-infection following a low dose (2 × 10^2^ tachyzoites). However, mice infected with PruΔ*gra86* demonstrated significantly increased survival (66.67%) compared to those infected with wild-type Pru (16.67%) (Figure 4C). A higher infection dose (5 × 10^4^ tachyzoites) led to all wild-type Pru-infected mice reaching humane endpoint by day 11, while 50% of mice infected with PruΔ*gra86* survived through day 30 (Figure 4D). These results suggest that GRA86 plays an important role in the virulence of the Pru strain.

Regarding brain cyst burden, surviving mice infected with 2 × 10^2^ wild-type Pru tachyzoites had an average of 91.7 ± 11.8 brain cysts per mouse, whereas no cysts were detected in mice infected with PruΔ*gra86* (Figure 4E). Successful infection in the *gra86* knockout group was confirmed by elevated serum IgG, IgG1, and IgG2a antibody levels compared to uninfected controls (Figure 4F). These findings demonstrate that GRA86 is important for the virulence and brain cyst formation of *T. gondii* Pru strain in vivo.

### 3.5. Deletion of gra86 Upregulates Host-Interaction Associated GRAs

**Figure 5:**
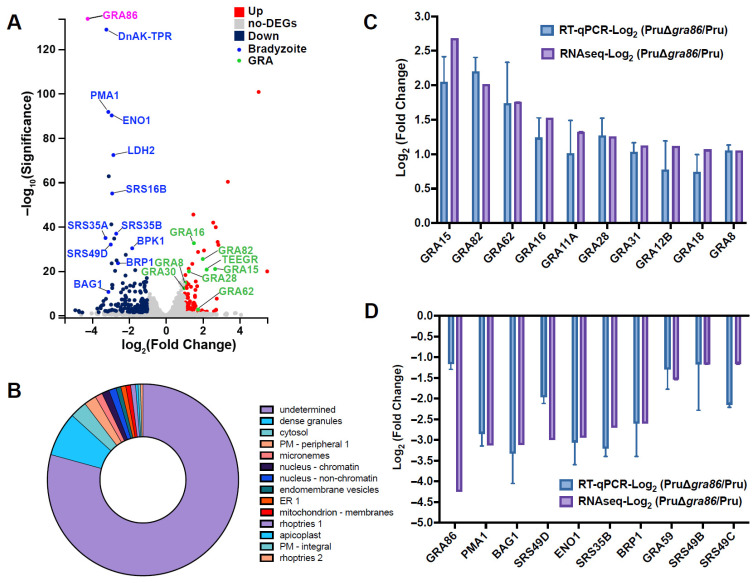
Transcriptional changes in *T. gondii* PruΔ*gra86* strain. (**A**) Volcano plot showing differentially expressed genes in PruΔ*gra86* versus wild-type Pru. (**B**) Predicted subcellular localization of proteins encoded by differentially expressed genes in PruΔ*gra86*. (**C**) RT-qPCR validation of upregulated GRAs in PruΔ*gra86*. (**D**) RT-qPCR validation of downregulated bradyzoite-associated genes in PruΔ*gra86*.

Transcriptomic analysis of intracellular *T. gondii* tachyzoites from PruΔ*gra86* and wild-type Pru strains revealed 241 differentially expressed genes, including 144 downregulated and 97 upregulated genes (Figure 5A, Supplementary Table S3). Most of the differentially expressed proteins were predicted to localize to dense granules, with 79.3% of proteins having unknown localization (Figure 5B). The number of upregulated GRAs surpassed downregulated ones (Supplementary Table S3). RT-qPCR validation confirmed the upregulation of several GRAs (Figure 5C), suggesting compensatory gene expression in the PruΔ*gra86* strain. Among the upregulated genes, several GRAs involved in host cell cycle modulation and immune response regulation, such as GRA15, GRA16, GRA18, and TEEGR, were significantly upregulated. These results indicate that GRA86 may play a key regulatory role in host–parasite interactions.

### 3.6. Deletion of gra86 Downregulates Bradyzoite-Associated Genes

Several genes, including *cst1* (cyst wall antigen), *mag1* (matrix antigen 1), and *bag1* (bradyzoite antigen 1), are specifically expressed during the bradyzoite stage of *T. gondii* [10]. RNA-Seq analysis revealed downregulation of key genes involved in bradyzoite development and maintenance, such as *bag1*, *brp1*, *eno1* (enolase-1), and *srs49c*, in the PruΔ*gra86* strain (Figure 5A and Supplementary Table S3). RT-qPCR validation confirmed the reduced expression of these bradyzoite-associated genes (Figure 5D), suggesting that GRA86 plays a role in promoting bradyzoite differentiation in *T. gondii*.

### 3.7. Loss of gra86 Impairs Bradyzoite Differentiation In Vitro

**Figure 6:**
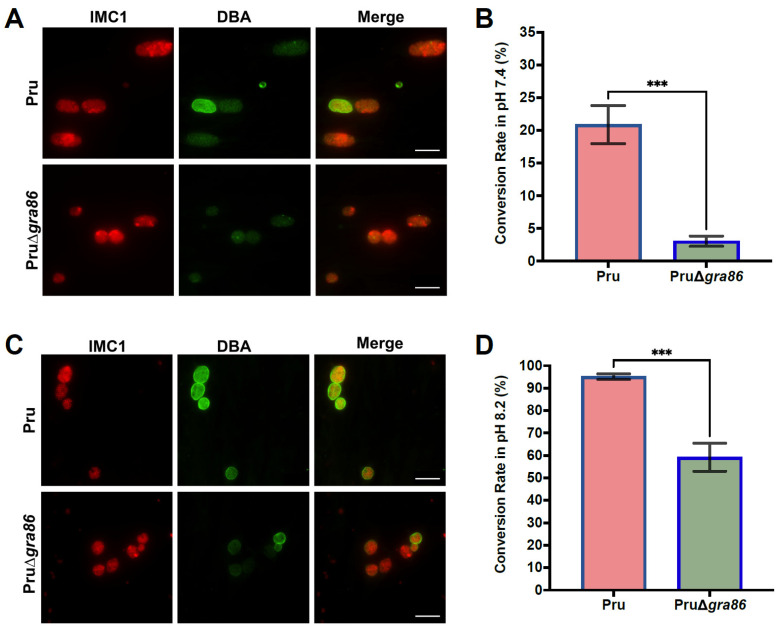
Loss of *gra86* significantly impairs in vitro bradyzoite conversion in *T. gondii* Pru strain. (**A**,**B**) Representative immunofluorescence images of vacuoles (**A**) and quantification of DBA-positive cysts (**B**) in Pru and PruΔ*gra86* strains under normal culture conditions (pH 7.4). (**C**,**D**) Representative images (**C**) and quantification of DBA-positive cysts (**D**) formed by Pru and PruΔ*gra86* strains under alkaline differentiation conditions (pH 8.2). Vacuoles were stained with DBA (green) and IMC1 antibody (red). Scale bar: 25 μm. Percentages of DBA-positive cysts were calculated by counting at least 100 vacuoles per sample from three independent experiments. *** *p* ≤ 0.001.

To assess the role of GRA86 in cyst differentiation, we compared the bradyzoite conversion rates of Pru and PruΔ*gra86* strains under normal and alkaline stress conditions. Our results revealed that PruΔ*gra86* tachyzoites exhibited significantly reduced bradyzoite conversion even under normal culture conditions (pH 7.4), consistent with RNA-Seq data (Figure 6A,B). Under alkaline stress (pH 8.2), the deletion of *gra86* further impaired cyst formation (Figure 6C,D). These findings demonstrate that GRA86 is important for bradyzoite differentiation in *T. gondii*.

## 4. Discussion

*Toxoplasma gondii* exhibits a remarkable ability to manipulate both its host cell environment and the host’s immune system to ensure survival and replication. This parasitic reprogramming is mediated by a range of secreted effectors, including GRAs and ROPs, which modulate various cellular processes such as nutrient uptake, cytoskeletal rearrangements, apoptosis inhibition, immune evasion, and gene expression alterations [12,14,39,56,57,58]. While considerable progress has been made in identifying and characterizing these parasite effectors, many of them, particularly the newly identified GRAs, such as GRA85–88, remain poorly understood in terms of their biological functions.

GRAs are synthesized in the endoplasmic reticulum, trafficked through the Golgi to dense granules, and subsequently transported to diverse subcellular localizations, where they perform a variety of roles [12,13]. For example, IVN-localized GRA2 and GRA6 are crucial for maintaining the integrity of the IVN and for host vesicle sequestration [59]. Other GRAs, including PVM-localized GRA17, GRA23, GRA47, and GRA72, regulate the permeability of the PVM [36,56,60], while GRA16 is exported to the host nucleus and modulates the host cell cycle [27,61]. Additionally, several GRAs, such as GRA7, GRA15, GRA24, TEEGR, and TgIST, are involved in immune modulation [18,28,62,63,64].

Our study revealed that GRA85–88 are primarily localized within the PV and co-localize with GRA12 in tachyzoites, supporting their role as dense granule-derived proteins and confirming previous spatial proteomics predictions [55]. Furthermore, these GRAs are expressed in bradyzoites and co-localize with both GRA12 and DBA, suggesting that they may contribute to cyst formation and maintenance, similar to other well-known cyst-related GRAs such as GRA12, GRA76, and CST2 [34,35,38,65].

Although the loss of *gra85*, *gra87*, or *gra88* did not impact in vitro growth or virulence, *gra86* deletion led to a significant attenuation of virulence in the Pru strain, a type II isolate, but not in the highly virulent RH strain. This strain-specific effect highlights the distinctive roles of GRAs in parasite virulence, and mirrors findings for other GRAs, such as GRA6 and GRA15, whose effects on host immune pathways also vary by strains [29,66]. These results identify GRA86 as an important virulence factor in *T. gondii* and suggest that its importance may be more pronounced under specific host–pathogen interactions or in particular strain backgrounds.

One of the hallmarks of *T. gondii* chronic infection is the formation of tissue cysts, particularly in the brain, muscles, and eyes [1]. Several GRAs, including GRA55, CST1, GRA76, and GRA50, are essential for cyst formation and maintenance [33,34,35,65]. In our study, the absence of GRA86 resulted in undetectable brain cyst burdens in mice, further underscoring the protein’s important role in cyst formation. In addition, in vitro experiments revealed a significant impairment in bradyzoite conversion in the PruΔ*gra86* strain, both under normal and alkaline stress conditions. Transcriptomic analysis further supported this finding, with a marked downregulation of bradyzoite-associated genes in the knockout strain. These results demonstrate that GRA86 is important for bradyzoite differentiation in *T. gondii*, both in vitro and in vivo. While our study provides compelling evidence for GRA86’s role in bradyzoite differentiation, the molecular mechanism by which it regulates this process remains unclear. Given that GRA86 lacks significant homology to known eukaryotic proteins, its precise function is still to be determined. Future studies focusing on domain mapping and interactome analyses using both knockout and complemented strains will be crucial for elucidating GRA86’s molecular role.

In addition to its role in cyst formation, the transcriptomic data also revealed an upregulation of several immune-modulatory GRAs in the PruΔ*gra86* strain, including GRA15, GRA16, GRA28, GRA18, and TEEGR. This result suggests that GRA86 may influence host–parasite interactions. Its loss could trigger a compensatory upregulation of other GRAs, thereby maintaining host–pathogen equilibrium. The absence of brain cysts following infection with PruΔ*gra86* suggests that the immune system may clear the infection more effectively in the absence of GRA86, preventing chronic infection establishment. Future research should aim to clarify the precise regulatory roles of GRA86 in modulating host immune pathways.

Immune-modulatory proteins regulate key host signaling pathways: GRA16 and TEEGR influence the host cell cycle [27,28], GRA15 activates NF-κB to induce pro-inflammatory cytokines [29], GRA18 promotes anti-inflammatory responses via β-catenin [67], and GRA28 modulates macrophage migration [68]. The upregulation of these effectors in the *gra86* knockout strain suggests potential functional redundancy within the GRA network, which could compensate for the loss of *gra86* under non-stress conditions. This redundancy may mask phenotypic severity in single-knockout models and complicate the assessment of GRA86’s full functional impact. Combinatorial knockout experiments targeting GRA86 and its upregulated counterparts will be essential for revealing the synergistic or compensatory roles of these effectors in chronic infection and immune evasion.

The trafficking of GRAs to their proper subcellular locations is critical for their function. GRAs such as the MYR complex and GRA45 mediate the translocation of effectors across the PVM [23,24,25,69], while GRA42, GRA43, and GRA72 facilitate PVM localization of GRA17 and GRA23 [70,71]. Given that some GRAs act as chaperones for the translocation of effectors [12], GRA86 could similarly facilitate the movement of effector proteins across the PVM, such as GRA16, GRA28, and TEEGR. Its loss may disrupt the localization of these effectors, leading to impaired immune modulation and reduced virulence. Future studies investigating the subcellular localization of these GRAs in *gra86* knockout parasites will be key to understanding their mechanistic role in *T. gondii* pathogenesis.

## 5. Conclusions

Our study identifies and characterizes four novel GRAs (GRA85–88) in *T. gondii*, which localize predominantly to the PV in tachyzoites and the cyst matrix in bradyzoites. While these proteins are dispensable for parasite growth in vitro, deletion of *gra86* specifically impairs virulence in the intermediate-virulent type II Pru strain but not in the highly virulent type I RH strain. Transcriptomic analysis of the PruΔ*gra86* strain reveals downregulation of bradyzoite-associated genes and upregulation of GRAs involved in host-parasite interactions. Importantly, the loss of *gra86* impairs both in vivo and in vitro bradyzoite differentiation. These findings highlight GRA86 as a notable regulator of chronic infection and provide new insights into the molecular mechanisms driving *T. gondii* development and pathogenesis. Future studies should focus on elucidating how GRA86 modulates bradyzoite differentiation and host immune responses, which may further advance our understanding of *T. gondii*’s ability to establish persistent infection.

## Figures and Tables

**Table 1:** Bioinformatic features of four novel dense granule proteins (GRAs) of *T. gondii*.

GRAs	Gene ID	Product Description	Predicted Location	Exons	Phenotype Value	Mol Wt (kDa)	Predicted Signal Peptide	TMHMM *
GRA85	TGME49_323110	Hypothetical protein	Dense granules	1	NA	36.315	no	yes
GRA86	TGME49_200360	Hypothetical protein	Dense granules	1	2.63	20.299	yes	no
GRA87	TGME49_306890	Hypothetical protein	Dense granules	1	1.78	147.459	no	no
GRA88	TGME49_266050	Hypothetical protein	Dense granules	1	2.1	27.276	no	no

* Prediction of transmembrane helices was performed using the TMHMM program version 2.0.

## Data Availability

All data generated or analyzed during this study are included in this published article and its Supplementary Materials. The raw RNA-Seq data have been deposited in the NCBI Sequence Read Archive (SRA) under the accession number PRJNA1305388. For additional information, please contact the corresponding authors.

## Supplementary Materials

The following supporting information can be downloaded at: https://www.mdpi.com/article/10.3390/ani15172591/s1. Table S1: Primers used for the construction of transgenic parasite strains; Table S2: Primers used in qRT-PCR experiments; Table S3a: Upregulated genes in Pru∆*gra86* strain; Table S3b: Downregulated genes in Pru∆*gra86* strain; Figure S1: Successful construction of four endogenously tagged strains (RH::GRA-HA); Figure S2: Successful construction of four *gra* gene knockout strains (PruΔ*gra*s).
