# Supplementary material for: GRA86 Is a Novel Dense Granule Protein Important for Virulence and Bradyzoite Differentiation in Toxoplasma gondii
Source: Animals (Basel). 2025 Sep 3;15(17):2591. doi: 10.3390/ani15172591 (PMC12427315; doi:10.3390/ani15172591)
Supplement: Supplementary file 1 [file animals-15-02591-s001.zip › Supplementary Figures/Figure S1.pdf]

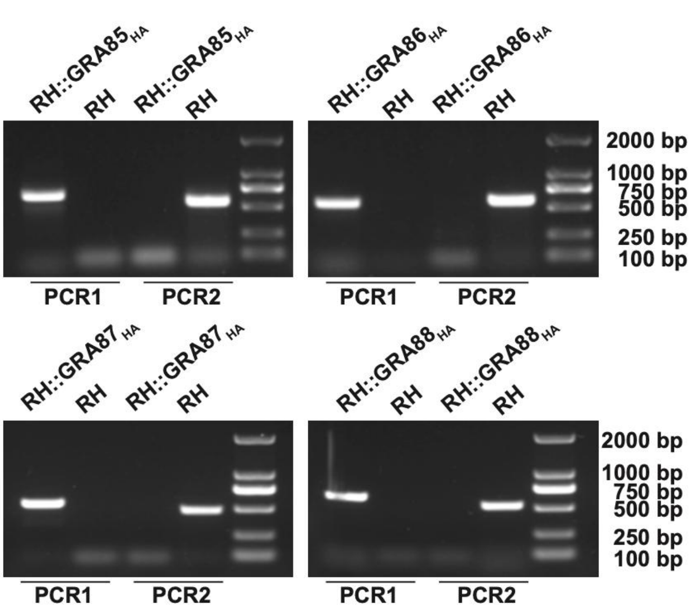

Figure S1. Validation of endogenously HA-tagged GRA85–88 strains in the RH background. PCR analysis confirming successful tagging of GRAs with a C-terminal 6×HA epitope. PCR1 detects the insertion of the HA tag near the stop codon of each *gra* gene. PCR2 verifies correct integration by amplifying the 3′ region replaced by the HA tag**.**
