# Supplementary material for: GRA86 Is a Novel Dense Granule Protein Important for Virulence and Bradyzoite Differentiation in Toxoplasma gondii
Source: Animals (Basel). 2025 Sep 3;15(17):2591. doi: 10.3390/ani15172591 (PMC12427315; doi:10.3390/ani15172591)
Supplement: Supplementary file 1 [file animals-15-02591-s001.zip › Supplementary Figures/Figure S2.pdf]

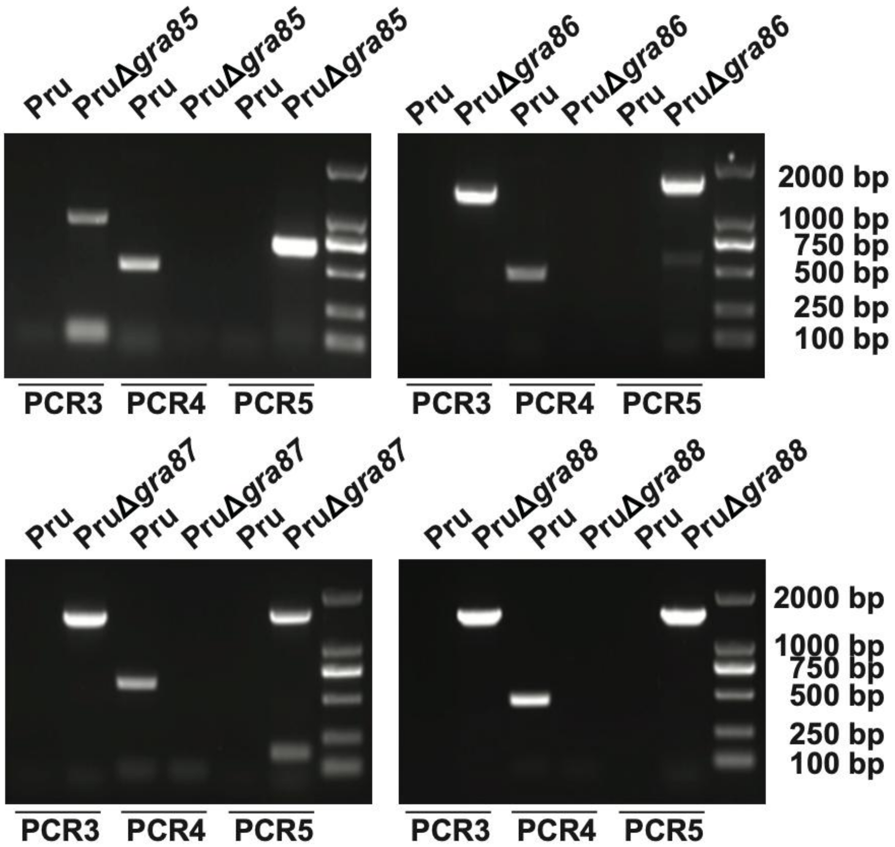

Figure S2. Generation and validation of four *gra* gene knockout strains (PruΔ*gra85–88*). PCR analysis confirming successful deletion of *gra85–88* in the type II Pru strain. PCR3 and PCR5 verify replacement of the 5′ and 3′ untranslated regions (UTRs), respectively, with the DHFR selection cassette. PCR4 confirms deletion of the *gra* coding sequences.
